# The Pattern Element Scale: A Brief Tool of Traditional Medical Subtyping for Dementia

**DOI:** 10.1155/2013/460562

**Published:** 2013-04-21

**Authors:** Jing Shi, Jinzhou Tian, Ziyi Long, Xiawei Liu, Mingqing Wei, Jingnian Ni, Jianping Liu, Tonghua Liu, Pengwen Wang, Hengge Xie, Bin Qin, Yongyan Wang

**Affiliations:** ^1^Third Department of Neurology, Dongzhimen Hospital, Beijing University of Chinese Medicine, Beijing 100700, China; ^2^Academic Department of Neurology, First School of Clinical Medicine, Beijing University of Chinese Medicine, Beijing 100700, China; ^3^Centre for Evidence-Based Chinese Medicine, Beijing University of Chinese Medicine, Beijing 100029, China; ^4^Beijing University of Chinese Medicine, Beijing 100029, China; ^5^Key Laboratory of Chinese Internal Medicine, Beijing University of Chinese Medicine, Ministry of Education, Beijing 100700, China; ^6^Department of Neurology, Chinese PLA General Hospital, Beijing 100853, China; ^7^Department of Neurology, Beijing Hospital, Beijing 100730, China; ^8^Institute of Clinical Medicine, China Academy of Chinese Medical Sciences, Beijing 100700, China

## Abstract

*Background*. Syndromes are defined by traditional Chinese medicine as consisting of different pattern elements. Few scales have been designed for differentiating pattern elements of dementia and have shown major flaws. Thus, a new pattern element scale (PES) was developed. This study aimed to evaluate the utility of the PES in dementia patients. *Methods*. A total of 171 dementia patients were enrolled, and their pattern elements were ascertained, first by clinicians using the PES, then compared with results by two experts to be used as a standard criterion independently. Reliability of the subscales of the PES was assessed by receiver operator characteristic curves. Correlations between the subscales of the PES and cognition were calculated by canonical correlation analysis. *Results*. The PES consisted of 11 pattern element subscales. The area under the curves of all subscales was 0.7 or above. Phlegm muddiness, blood stasis, and yang hyperactivity subscales showed optimal sensitivity and specificity in discriminating pattern elements. Other subscales showed relatively lower sensitivity but higher specificity. Memory and language were significantly correlated to *qi* deficiency and the blood stasis pattern element. *Conclusion*. The PES can accurately and easily discriminate pattern elements and is a helpful tool for traditional medical subtyping of dementia.

## 1. Introduction

Alzheimer's disease (AD) is a progressive and irreversible neurodegenerative disorder that leads to cognitive and behavioral impairments. As shown in previous studies, AD constitutes 60 to 80% of all cases of dementia [1]. The ultimate aim of AD therapy is to stop or slow down the disease progression. However, cholinesterase inhibitors and memantine, the currently available *N*-methyl-D-aspartate receptor antagonist, have a modest clinical effect on the symptoms and do not prevent the deterioration associated with dementia [2, 3]. Therefore, finding an effective method to treat AD still poses a significant clinical challenge [4].

Herbal medicine has long been used in a traditional form in China as therapy for dementia. *The Complete Work of Jingyue*, published in 1624, contains the earliest known description in the world of an herbal therapeutic strategy for dementia [5]. Following that text, *The Records for Syndromes' Differentiation*, published in 1687, develops herbal remedies for dementia [6]. However, according to the principle of the use of herbal therapy in the traditional form, a syndrome defined by traditional Chinese medicine (TCM) is the basis for prescribing a relevant treatment. In other words, the determination of treatment is based on the differentiation of syndromes. The syndrome consists of different pattern elements, such as phlegm muddiness, blood stasis, endogenous heat, *yin* deficiency, *qi* deficiency, *yang* hyperactivity, and marrow deficiency. Each pattern element has its own clinical features, which are usually abstracted from medical history, symptoms, and tongue-pulse signs of the disease. Because there is no recognized standard method to discriminate the pattern elements, it is difficult to compare treatment effects between different studies.

At present, there are few scales specifically designed for the research of discriminating syndromes in dementia patients, and these scales have major flaws [7–9]. First, the operation of these scales is clinically inconvenient. Second, many scales were developed based on expert consensus only. Third, the validity and reliability of all of these scales have not been evaluated in patients with dementia, and their usage needs further validation. Given that the amount of research using Chinese medicine with patients with dementia has accelerated in recent years and is likely to increase, the need for a new pattern element scale (PES) specific to dementia is apparent. We designed a PES for this purpose [10, 11]. 

Initially the PES was composed of 11 pattern element subscales, including phlegm muddiness, kidney deficiency, spleen deficiency, blood stasis, *qi* deficiency, *yin* deficiency, *yang* deficiency, blood deficiency, marrow deficiency, endogenous heat, and *yang* hyperactivity (See the appendix). Each subscale contains seven items, and every pattern element subscale has a maximum score of 30 points. When the score was 6 points and above, it was considered to efficiently diagnose this pattern element of each subscale. The subscales rate the major pattern elements of dementia, and the PES can be completed in a relatively short period of time. This study aimed to provide further information about the reliability and parallel validity of the PES for discriminating the pattern elements in people with dementia.

## 2. Methods

### 2.1. Participants

A total of 171 patients with dementia, including AD and vascular dementia (VaD), were enrolled from Dongzhimen Hospital, Daxing District Psychiatric Hospital and three communities around Dongzhimen Hospital, in Beijing, China, between April 2011 and February 2012.

The diagnosis of dementia was based on the *Diagnostic and Statistical Manual of Mental Disorders*, fourth edition [12]. The following criteria were used to define dementia: (1) minimental state examination (MMSE) cut-off scores: ≤19 for illiteracy, ≤22 for primary school, ≤23 for middle school, and ≤26 for higher education [13]; (2) two or more domains of cognitive impairment; (3) continued aggravation of memory and other cognitive functions; (4) absence of conscious disturbance; (5) impaired activities of daily living (ADL score ≥16) [14]; (6) clinical dementia rating score (CDR) ≥0.5 [15]; (7) a score of ≤12 on the Hamilton depression scale (HAMD for 17 items) [16]; and (8) exclusion of other diseases that may cause cognitive impairment such as DSM-IV-defined psychosis, major depression, bipolar disorder, or alcohol or substance abuse. The diagnosis of AD was based on the National Institute of Neurological Communicative Disease and Stroke and Alzheimer's Disease and Related Disorders Association (NINCDS and ADRDA) criteria [17], and the diagnosis of VaD was based on the National Institute of Neurological Disorders and Stroke and Association Internationale pour la Recherche et l'Enseignement en Neurosciences (NINDS and AIREN) [18].

### 2.2. Procedure

**Figure 1:**
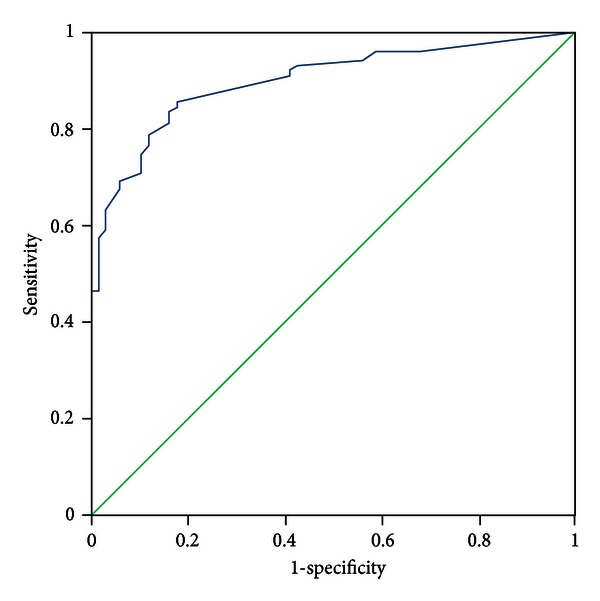
ROC curve of subscale of phlegm muddiness.

**Figure 2:**
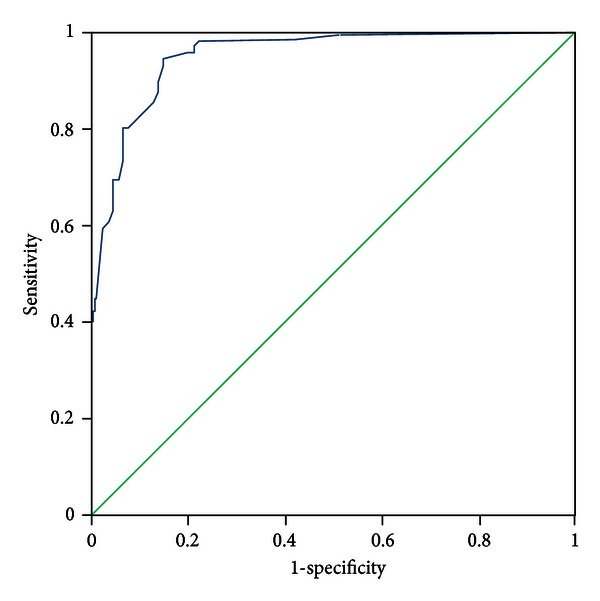
ROC curve of subscale of blood stasis.

**Figure 3:**
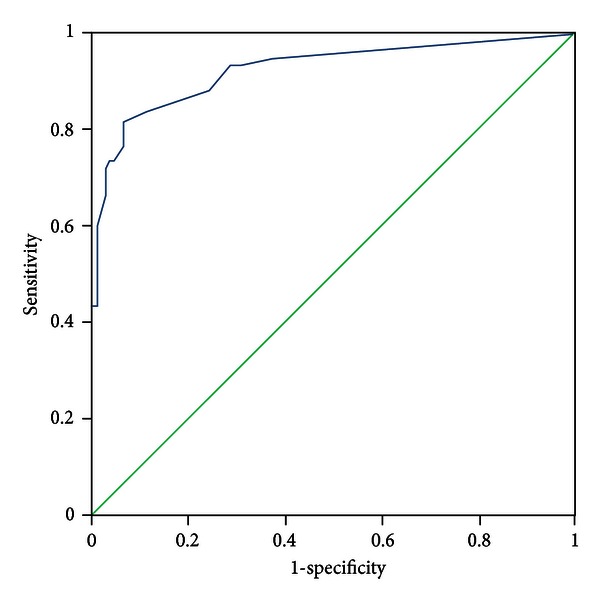
ROC curve of subscale of *yin* deficiency.

**Figure 4:**
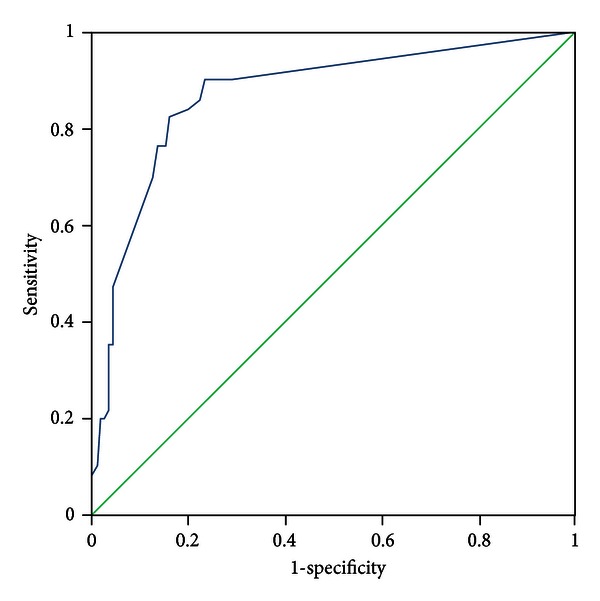
ROC curve of subscale of *qi* deficiency.

**Figure 5:**
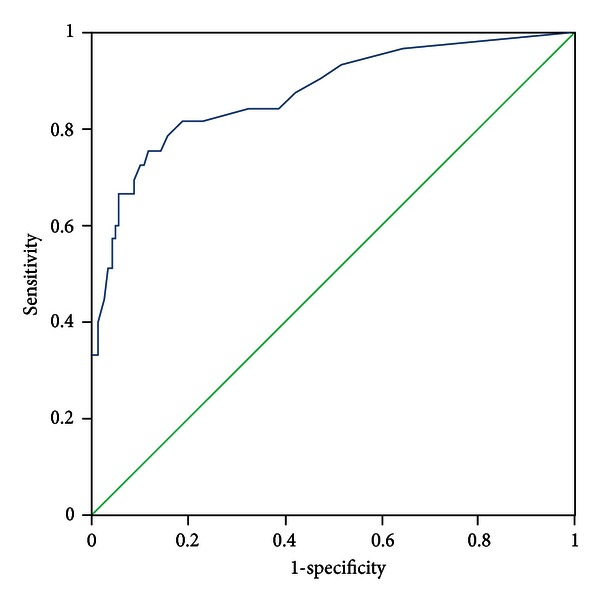
ROC curve of subscale of marrow deficiency.

**Figure 6:**
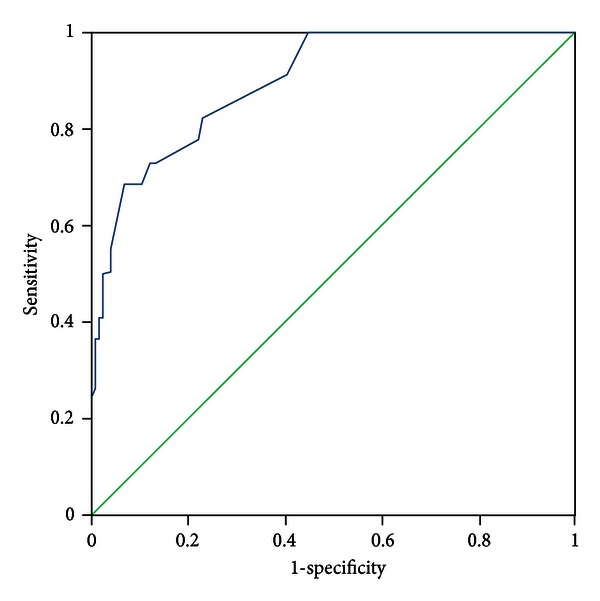
ROC curve of subscale of endogenous heat.

**Figure 7:**
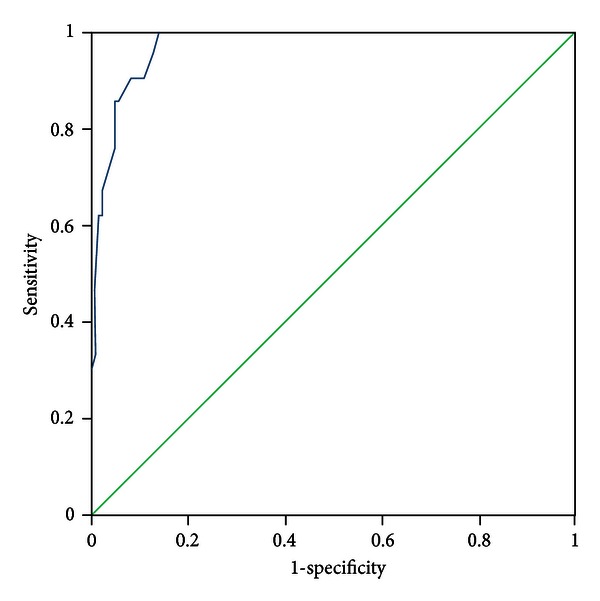
ROC curve of subscale of *yang* hyperactivity.

**Figure 8:**
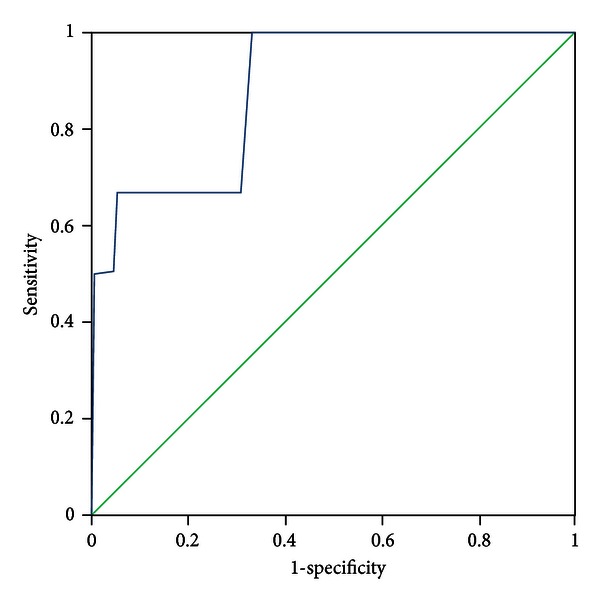
ROC curve of subscale of *yang *deficiency.

**Figure 9:**
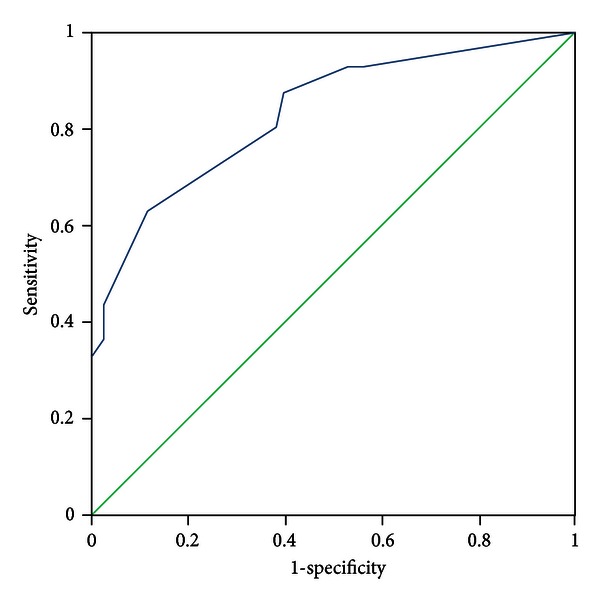
ROC curve of subscale of spleen deficiency.

**Figure 10:**
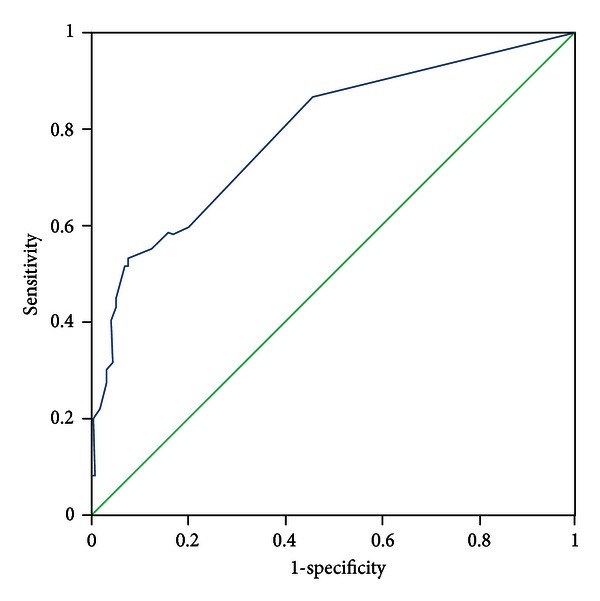
ROC curve of subscale of kidney deficiency.

**Figure 11:**
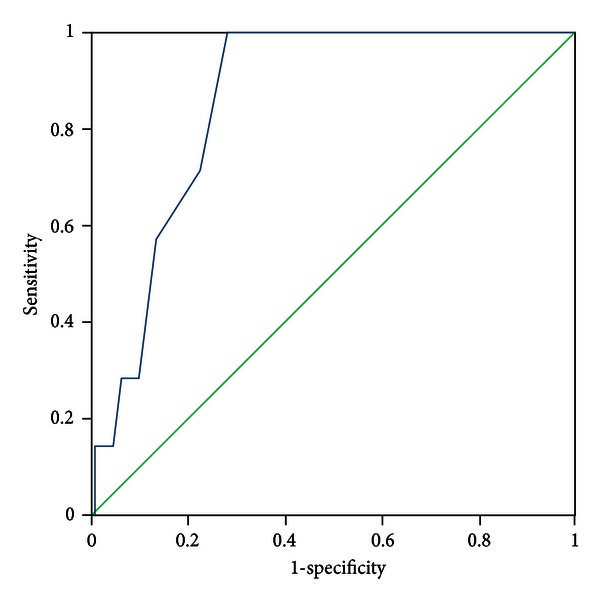
ROC curve of subscale of blood deficiency.

All patients were evaluated by two experts who discriminated the pattern elements independently. In the case of discrepancies in the evaluation of pattern elements, a third expert was consulted, the pattern elements were discussed, and a final decision was made. The diagnosis by the experts was recognized as a standard criterion. The PES was administered to all participants by five trained clinicians who discriminated pattern elements according to authors' instructions. The five clinicians were blinded to the pattern elements that were discriminated by experts. Sensitivity and specificity were evaluated with experts' diagnosis as the standard criterion using the receiver-operator characteristic (ROC) curves for each subscale, respectively (see Figures 1–11). 

### 2.3. Statistical Analysis

The statistical analyses were conducted using SPSS 18.0 for Windows. The ROC curves were calculated by plotting the sensitivity against the 1-specificity for each subscale. In addition, the positive predictive value (PPV) and negative predictive value (NPV) were calculated based on the prevalence among the selected sample.

The parallel validity between the judgment of the experts and discrimination of pattern elements with the PES was also calculated by kappa value using the chi-square test. The correlations between scores of the subscales (including 11 pattern elements) of the PES and cognition score as measured by MMSE (including time orientation, place orientation, memory, calculation/attention, recall, and language) were calculated using canonical correlation analysis.

This study was undertaken in accordance with the principles of the Declaration of Helsinki. The protocol was approved by the Dongzhimen Hospital Institutional Ethics Committee. The patients and their caregivers provided written informed consent.

## 3. Results

### 3.1. Demographic Characteristics

A total of 171 participants of dementia were enrolled in this study. The sample included 92 (54%) participants with probable AD, 32 (19%) with a diagnosis of probable VaD, 40 (23%) with mixed dementia, and 7 (4%) with other dementia. The sample was composed of 85 males and 86 females. The mean age was 73.0 ± 8.8 years old. The mean year of formal education was 9.07 ± 4.52. The mean MMSE score was 16.25 ± 6.20, and the severity of dementia was as follows: 59 (34.5%) with mild dementia, 57 (33.3%) with moderate dementia, and 55 (32.2%) with severe dementia.

### 3.2. Sensitivity and Specificity of Subscale in PES

Tests for sensitivity and specificity of each subscale were demonstrated, respectively, in Table 1. The area under the curve (AUC) was above 0.7 for each subscale (Table 2); the specificity was relatively high (>80%) except for the kidney deficiency subscale. When the cut-off score was set at 6, the phlegm muddiness, blood stasis, and *yang* hyperactivity showed an optimal sensitivity, with 83.5%, 94.7%, and 90.5%, respectively, and the specificity was 83.8%, 85.3%, and 92.0%, respectively, for discriminating pattern elements. The sensitivity of *yin* deficiency, *yang* deficiency, *qi* deficiency, marrow deficiency, and blood deficiency subscales was poor (58.8%–68.3%). More interestingly, the specificity of these subscales was excellent (>90%), which suggests a lower false positive rate. It indicates that these subscales have a lower capability to discriminate those pattern elements. The subscales of endogenous heat and blood deficiency showed acceptable sensitivity, 72.7% and 71.4%, respectively, and the specificity was relatively high (87.9% and 80.4%, resp.). 

PPV and NPV were calculated based on the baseline rate of each pattern element of the sample (Table 1). The NPVs of all subscales were good to excellent (72.6%–98.6%). The PPVs were much higher in the phlegm muddiness, blood stasis, *yin* deficiency, *qi* deficiency, and marrow deficiency, with each higher than 75.0%; however, the other six subscales showed much lower PPVs (11.9%–65.2%). 

 The kappa values are shown in Table 1. The subscale of blood stasis had good parallel validity (kappa = 0.79), while the subscales of kidney deficiency, spleen deficiency, and blood deficiency had much poorer validity. The other subscales showed a medium parallel validity (kappa values between 0.53 and 0.69). 

### 3.3. Correlation between Subscales of the PES and MMSE

As shown in Tables 3, 4, and 5, we calculated the correlations between the scores of the subscales from the PES with the cognition score of the MMSE using canonical correlation analysis. There was a significant correlation between the subscales in the PES and cognition scores (*r* = 0.507, *P* < 0.001). Further analysis showed that memory and language were significantly negatively correlated with *qi* deficiency and blood stasis, which indicated that higher scores on the *qi* deficiency and blood stasis subscales are associated with severe impairment of memory and language. 

## 4. Discussion

At present, there are three widely used differentiating scales of traditional syndromes for dementia in China. Fu [7] published a typing scale of six pattern elements for dementia in 1991 including AD, VaD, mixed dementia, Pick's disease, and other conditions. It was the first subtyping scale for dementia and was widely used in the field of TCM. However, the clinical features of each element in Fu's scale were described by narrative language, without detailed scoring instructions. Thus, Fu's scale appeared to be operated with difficulty by different clinicians. Tian et al. [8] published a scale for the differentiation of syndromes of vascular dementia (SDSVD) in 2000. The SDSVD is a seven-element scale for vascular dementia, and each element subscale is composed of several brief scored items that include symptoms and pulse-tongue signs. The total score of each element subscale is 30, and the cut-off of each subscale is 7 points. Mild dementia was determined with a score of ≥7 and ≤14, moderate dementia with a score of ≥15 and ≤22, and scores of ≥23 were defined as severe dementia. The SDSVD is practical because of the clear factor items and cut-off scores. However, the SDSVD was designed for VaD only, and its applicability in other dementia patients is still questionable. Zheng [9] published a five-element scale for senile dementia in 2002. The clinical features of each element were divided into primary and secondary symptoms, and the diagnosis is based on the number of primary and secondary symptoms added together. Zheng's scale is easy to operate because of the simple diagnostic criteria. However, the description of the symptoms is not standard; thus, it is easy to cause misinterpretations between different investigators. Additionally, the severity of the symptoms has not been quantified in Zheng's scale.

Given that early epidemiological studies showed that VaD was the main cause of dementia in China, and the prevalence of VaD was much higher than AD [19–21], researchers in China tended to focus on syndromes of VaD over the past decade. However, recent studies have shown a tendency toward change, with AD as the main subtype of dementia in China [22–24]. To adapt to this change, further studies about syndromes of AD should be developed. The data source of the PES was mostly AD and VaD patients recruited for this study; thus, it could be said that the subscales developed in this study are suitable for both AD and VaD. 

The findings of the current study demonstrated that the 11 subscales of the PES showed high reliability for discriminating pattern elements. The AUC for each of the subscales was higher than 0.7 and achieved the minimum requirements for diagnostic tests [25]. The AUC for the phlegm muddiness, blood stasis, *yang* hyperactivity, and *yin* deficiency subscales was higher than 0.9, which showed an excellent capacity to discriminate pattern elements. The subscales for phlegm muddiness, blood stasis, and *yang* hyperactivity showed a high degree of sensitivity (>83.5%), and the specificity was excellent (>83.8%); therefore, those three subscales are suitable for case screening in epidemiological surveys and strict inclusion criteria in clinical investigations. The sensitivity of the *yin* deficiency, *yang* deficiency, *qi* deficiency, marrow deficiency, and spleen deficiency subscales was poor (58.1%–68.3%) but with higher specificity (>80.0%); these subscales are also suitable for strict inclusion criteria in clinical investigations.

The subscale for blood stasis had a good parallel validity (kappa = 0.79), while the subscales for kidney deficiency and spleen deficiency had much poorer validity (0.248–0.273). The other subscales showed a medium parallel validity (kappa value between 0.4 and 0.7). 

The PPVs and NPVs were related to the baseline rate of prevalence; higher prevalence may induce relatively lower PPVs, and higher prevalence may induce relatively lower NPVs [26]. Because of a lower prevalence in this sample, *yang* deficiency, spleen deficiency, blood deficiency, and endogenous heat showed relatively lower PPVs. The phlegm muddiness and kidney deficiency showed relatively lower NPVs. The possible reason may be a result of the high prevalence. 

Studies have reported that memory functions are correlated with kidney essence vacuity and turbid phlegm blocking the upper orifice in mild cognitive impairments and AD patients. Turbid phlegm blocking the upper orifice was correlated with executive function and global cognition, as measured by the MMSE in VaD patients. There are many treatments in Chinese medicine for replenishing kidney essence or removing phlegm, which can improve memory and cognitive function [27–29]. The present study showed that deficiency of *qi* and blood stasis was negatively correlated to memory and language, indicating that higher scores related to deficiency of *qi* and blood stasis are associated with more severe impairment of language and memory. The different results may be because the scores in previous studies were calculated based on dementia and nondementia, and the result was the correlation between the pattern elements and the disease. In the present study, the correlations were based only on scores from patients with dementia, and the result was the correlation between the severity of cognition and pattern elements in dementia patients. This result indicated that scores on the subscales of the PES have an inherent correlation with the objective scale examination of dementia. Therefore, pattern elements were not only correlated with disease but were also correlated with cognition in the same patients. 

There are limitations in the present study. First, there were no normal controls or patients with mild cognitive impairment recruited in the present study; thus, differences in the pattern elements distributions of different groups were unclear. Second, because of time limits, the test-retest reliability, the internal consistency between clinical feature items, and the cut-off scores for different levels of severity (mild, moderate, and severe dementia) of the subscales were not determined. More participants and followup tests are needed in future studies.

In summary, the present study shows that the PES, a brief tool for traditional medical subtyping for dementia, is helpful to accurately and easily discriminate pattern elements. From this scale, the phlegm muddiness, blood stasis, and *yang* hyperactivity subscales were shown to be superior to other subscales in terms of parallel validity and diagnostic properties. 

## Figures and Tables

**Table 1:** Parallel validity and reliability of subscales in pattern element scales.

Pattern element	Parallel validity			Diagnostic reliability			
	Kappa value	Pearson chi-square	*P* value	Sensitivity	Specificity	PPV	NPV
Kidney deficiency	0.273	63.9	0.000	65.9%	71.2%	65.2%	72.6%
Spleen deficiency	0.248	125.5	0.000	58.1%	92.8%	47.3%	84.8%
*Qi* deficiency	0.538	50.9	0.000	58.8%	91.7%	75.0%	84.0%
*Yin* deficiency	0.699	87.7	0.000	68.3%	97.3%	93.2%	85.0%
*Yang* deficiency	0.591	86.8	0.000	63.8%	92.3%	76.0%	84.7%
Marrow deficiency	0.621	66.7	0.000	63.6%	94.9%	75.0%	91.6%
*Yang* hyperactivity	0.685	84.4	0.000	90.5%	92.0%	61.3%	98.6%
Endogenous heat	0.492	44.2	0.000	72.7%	87.9%	47.1%	95.6%
Phlegm muddiness	0.663	75.6	0.000	83.5%	83.8%	88.7%	77.0%
Blood stasis	0.790	108.0	0.000	94.7%	85.3%	83.7%	95.3%
Blood deficiency	0.547	73.913	0.000	71.428%	92.4%	75.1%	84.1%

PPV: positive predictive value; NPV: negative predictive value.

**Table 2:** Area under curves of subscales in the pattern element scale.

Pattern element	Area	Standard error	Progressive sig.	Progressive 95% confidence interval	
				Lower limit	Upper limit
Kidney deficiency	0.705	0.040	0.000	0.645	0.785
Spleen deficiency	0.700	0.062	0.040	0.583	0.770
*Qi* deficiency	0.873	0.031	0.000	0.811	0.934
*Yin* deficiency	0.927	0.023	0.000	0.881	0.972
*Yang* deficiency	0.833	0.012	0.000	0.852	0.946
Marrow deficiency	0.877	0.037	0.000	0.804	0.949
*Yang* hyperactivity	0.974	0.011	0.000	0.952	0.996
Endogenous heat	0.897	0.033	0.000	0.833	0.961
Phlegm muddiness	0.900	0.023	0.000	0.855	0.946
Blood stasis	0.955	0.014	0.000	0.928	0.982
Blood deficiency	0.869	0.032	0.000	0.807	0.946

Under nonparametric hypothesis, null hypothesis: actual area is 0.5.

**Table 3:** The canonical correlation coefficient and *F*-test between score of subscales in PES and cognition measures of MMSE.

Canonical variable	Canonical correlations	Wilk's lambda	Chi-SQ	DF	Sig.
1	0.507	0.592	85.433	42	0.000
2	0.327	0.797	37.041	30	0.176
3	0.267	0.892	18.562	20	0.55
4	0.152	0.961	6.463	12	0.891
5	0.123	0.984	2.632	6	0.853
6	0.028	0.999	0.132	2	0.936

MMSE: minimental state examination; PES: pattern element scale.

**Table 4:** Cross loadings for set-1.

Original variable	1	2	3	4	5	6
Time orientation	0.32	−0.177	−0.04	0.06	0.027	0.008
Place orientation	0.319	−0.088	−0.19	0.014	0.003	−0.004
Memory	0.416*	0.025	−0.071	0.018	0.035	−0.011
Recall	0.124	0.08	−0.116	0.086	0.029	0.016
Attention/calculation	0.191	−0.067	−0.067	0.095	−0.045	−0.014
Language	0.449*	0.011	−0.097	0.018	−0.032	−0.002

*Means *P* ≤ 0.05.

**Table 5:** Cross loadings for set-2.

Original variable	1	2	3	4	5	6
Phlegm muddiness	0.127	0.229	−0.099	0.022	0.043	0.002
Blood stasis	−0.17*	−0.11	−0.051	−0.054	0.096	0.002
*Yin* deficiency	−0.089	−0.07	−0.066	−0.02	−0.058	−0.022
*Qi* deficiency	−0.377*	0.015	0.041	0.051	−0.01	−0.001
Marrow deficiency	−0.016	−0.12	−0.126	−0.062	−0.074	0.003
Endogenous heat	−0.053	0.04	0.08	−0.114	−0.034	−0.014
*Yang* hyperactivity	0.129	−0.141	0.043	−0.017	−0.017	−0.021
Kidney deficiency	−0.053	−0.024	−0.01	−0.055	0.004	0.003
Spleen deficiency	−0.061	−0.03	−0.016	−0.006	−0.003	0.002
Blood deficiency	0.13	−0.13	0.040	−0.016	−0.015	−0.022
*Yang* deficiency	0.125	0.250	−0.011	0.020	0.041	0.003

*Means *P* ≤ 0.05.

**Table 6:** 

Number	Pattern element	Clinical feature item	Weight score
		Limp and aching lumbar	5
		Urinary incontinence	5
		Deafness	4
1	Kidney deficiency	Sexual hypoactivity	4
		Frequent micturition with clear urine	3
		Dizziness and tinnitus	2
		Thready and weak pulse	1
	**Total**		**24**

		Decrease of diet and anorexia/reduced appetite	4.5
		Involuntary drooling	4
		Sloppy stool	3.5
2	Spleen deficiency	Weakness of four extremities	3
		Decrease of diet and abdominal distension	3
		Pale tongue with indentation on margin	3
		Slow pulse	2
	**Total**		**23**

		Shortage of breath and disinclination of talking	5
		Spontaneous perspiration	5
		Palpitation	4
3	*Qi* deficiency	Hypodynamia	3
		White and lusterless complexion	3
		Hyperarousal and timidity	2
		Thready pulse	2
	**Total**		**24**

		Sallow complexion	5
		Pale tongue and lip	4
		Dizziness	3.5
4	Blood deficiency	Palpitation	3
		Ennui and hypodynamia	2
		Numbness of extremities	2
		Thready pulse	2
	**Total**		**21.5**

		Red and dry tongue	5
		Thin tongue fur	4.5
		Dry eyes	4.5
5	*Yin* deficiency	Emaciation	4.5
		Thready and rapid pulse	3
		Dry stool	3
		Night sweat	3
	**Total**		**27.5**

		Chilly	5
		Cold limbs	4
		Diarrhea almost every morning around three to five o'clock	4.5
6	*Yang* deficiency	Difficulty in micturition	3.5
		Blue and purple lip or nail	3
		Pale tongue with watery tongue fur	3
		Weak and sunken pulse	2
	**Total**		**25**

		Dysbasia	5.5
		Drumble/bradyphagia	5
		Aching limbs and tibia	5
7	Marrow deficiency	Loss of tooth/teeth	4.5
		Thin pale tongue	3.5
		Dry and withered hair	3.5
		Somnolence	2
	**Total**		**29**

		Irritable mood and irascibility	5.5
		Dizziness and vertigo	5
		Tremoring tongue	4.5
8	*Yang* hyperactivity	Subsultus tendinum or jumping of muscle	4
		Tinnitus with a loud wavelike sound	4
		Headache	4
		Wiry pulse/stringy pulse	3
	**Total**		**30**

		Halitosis	5
		Yellow tongue fur	5
		Red complexion	4
9	Endogenous heat	Red tongue	4
		Constipation with dry stool	2.5
		Bitter taste in mouth	2
		Rapid pulse	2
	**Total**		**24.5**

		Excessive phlegm	5
		Greasy and turbid tongue fur	3
		Enlarged tongue	2.5
10	Phlegm muddiness	Nausea	3
		Distension and fullness in chest and stomach	3
		Slippery pulse	3
		Dizziness and demented/madness	2
	**Total**		**21.5**

		Petechia in tongue	6
		Dark purple tongue	5
		Absurd and bizarre thoughts	4
11	Blood stasis	Dark purple lip	3.5
		Grayish complexion	3.5
		Stabbing pain or oppressive pain	3
		Rough pulse	2
	**Total**		**27**

## Appendix

### The Pattern Element Scale for Dementia

See Table 6.
